# Formation of Rutin–β-Cyclodextrin Inclusion Complexes by Supercritical Antisolvent Precipitation

**DOI:** 10.3390/polym13020246

**Published:** 2021-01-13

**Authors:** Paola Franco, Iolanda De Marco

**Affiliations:** Department of Industrial Engineering, University of Salerno, Via Giovanni Paolo II, 132, 84084 Fisciano (SA), Italy; pfranco@unisa.it

**Keywords:** SAS precipitation, β-cyclodextrin, rutin, inclusion complex, supercritical carbon dioxide

## Abstract

In this work, rutin (RUT)–β-cyclodextrin (β-CD) inclusion complexes are prepared by Supercritical AntiSolvent (SAS) precipitation. Well-defined composite microparticles are obtained at guest:host ratios equal to 1:2 and 1:1 mol:mol. The dimensions of composite particles range between 1.45 ± 0.88 µm and 7.94 ± 2.12 µm. The formation of RUT–β-CD inclusion complexes has been proved by different analyses, including Fourier transform infrared spectroscopy, Differential Scanning Calorimetry, X-ray diffraction, and UV-vis spectroscopy. The dissolution tests reveal a significant improvement in the release rate of RUT from inclusion complexes. Indeed, compared to the unprocessed RUT, the dissolution rate is about 3.9 and 2.4 times faster in the case of the complexes RUT–β-CD 1:2 and 1:1 mol:mol, respectively. From a pharmaceutical/nutraceutical point of view, CD-based inclusion complexes allow the reduction of the polymer amount in the SAS composite formulations.

## 1. Introduction

Rutin (RUT) is a flavonoid, which is a group of polyphenols, also defined as vitamin P. RUT is also known as quercetin-3-rhamnosyl glucoside since it is the most common and widespread glycoside form of quercetin [1]. As most of the flavonoids, RUT is present in a variety of vegetables, fruits, and plants [2,3,4,5]. This natural compound offers numerous benefits for human health, including antioxidant, anti-inflammatory, antiviral, antidiabetic, and anticancer activities [6,7,8,9]. It is also useful in the treatment of cardiovascular diseases, venous insufficiency, and capillary impairment [9]. In particular, these beneficial properties of RUT can be exploited both to treat and prevent cancer, cardiovascular, pancreas, and liver diseases. RUT is included in a wide number of supplements, multivitamin preparations, and herbal remedies. It is also widely spread in cosmetics, due to its action against hair follicle degeneration (preventing the causes of baldness) and to the benefits provided to the skin; i.e., the promotion of collagen synthesis, the sunscreen effect and the anti-fatigue activity [9,10].

However, during processing and storage, external factors (e.g., oxygen, possible changes in the temperature and pH) can cause chemical changes, oxidation, or even RUT degradation. Moreover, like almost all flavonoids, it is poorly-water soluble, resulting in a low bioavailability [1,11].

Despite the truly valuable properties of RUT in the pharmaceutical and nutraceutical fields, few studies have been conducted to date to increase RUT dissolution rate and, consequently, its bioavailability [12,13,14,15]. For this purpose, coprecipitated microparticles [15,16] or inclusion complexes [12,13,14,17] consisting of a hydrophilic polymer and the active principle are produced.

The attainment of inclusion complexes is based on the use of cyclodextrins (CDs), cyclic oligosaccharides, used in the nutraceutical and pharmaceutical fields to stabilize and protect several active compounds, to mask their unpleasant odor/taste, as well as to increase the drug dissolution rate [18,19,20].

CDs are characterized by a truncated cone-like structure: The hydrophilic external surface entails the water solubility of CDs, whereas the hydrophobic internal cavity allows them to incorporate different molecules with proper size, shape, and hydrophobicity [21]. As a consequence, guest/host inclusion complexes are formed through non-covalent interactions, such as hydrogen bonds, hydrophobic bonds, and van der Waals forces. Among the various CDs, β-cyclodextrin (β-CD) is the most commonly used one, because α-cyclodextrin has a cavity too small to encapsulate the majority of active molecules, whereas γ-cyclodextrin is too expensive [19].

In some papers, the preparation of RUT–CD inclusion complexes was attempted by using traditional technologies; i.e., kneading [12,13], co-grinding [14], coevaporation [13], and precipitation [17] methods. In these works, the formation of RUT/CD complexes was proved through some analytical techniques, including Fourier transform infrared spectroscopy (FT-IR) and differential scanning calorimetry (DSC) analyses [13,14,17]. Sri et al. [13] compared two different methods to obtain RUT–CD inclusion complexes, to increase the active principle dissolution rate. In some cases, RUT release kinetics from complexes were not satisfactory. For example, in the study of Paczkowska et al., it was noted that only 60% of RUT was released from inclusion complexes after 50 h [14]. Probably, this result can be ascribed to the morphology of the composite powders; i.e., very large crystals, as observed using a field emission scanning electron microscope. In another paper [17], the dissolution tests were not even performed. In summary, the papers focused on obtaining inclusion complexes containing rutin are not exhaustive and fully satisfactory; this can be linked to the drawbacks of the used traditional techniques: multistage processing, possible thermal degradation of the encapsulated active principle, high solvent residues in the product, and attainment of composites with irregular morphology/shape [14,22,23,24]. These limits can be overcome by employing supercritical carbon dioxide (scCO_2_)-based technologies [25,26,27]; the various techniques can be classified according to the role played by scCO_2_ with respect to the active principle. One of the most employed is the Supercritical AntiSolvent (SAS) technique, in which the scCO_2_ is used as the antisolvent [28,29]. The process is based on two prerequisites: the miscibility of scCO2 with the organic solvent at the process conditions and the insolubility of the solute that has to be micronized in the mixture formed by the solvent and scCO2. Considering that the diffusivity of carbon dioxide at supercritical conditions is comparable to the one of a gas, scCO_2_ rapidly diffused into the liquid droplet, inducing the solute supersaturation and its subsequent precipitation.

SAS coprecipitation is generally effective when microspheres consisting of the active principle dispersed in one of the polymers generally used as carriers for this type of process are obtained [30]. Up to now, the bioavailability of various active compounds was significantly improved by the SAS process. Nevertheless, one of the limitations of the SAS process is the use of high polymer contents to ensure the formation of composite microparticles with an increased drug dissolution rate [15,30]. In order to give a striking example, it is possible to consider the results obtained in the work of Ozkan et al. [15], in which, through the SAS process, composite microparticles containing rutin using polyvinylpyrrolidone (PVP) as the carrier, were obtained using RUT:polymer ratios equal to 1:20 and 1:10 *w*:*w*. These are the typical polymer/drug ratios using PVP as the carrier which, to date, is the most used polymer for SAS coprecipitation [30].

A possible solution that aims at the coprecipitation with a lower quantity of polymeric carrier consists of the use of CDs. However, the SAS technique was applied to prepare CD-based inclusion complexes in a limited number of literature studies [20,31,32,33,34,35,36]. Moreover, in none of them, the preparation of inclusion complexes containing RUT was attempted, despite the numerous benefits offered by RUT for human health. In almost all these works focused on the application of the SAS technique to prepare complexes, hydroxypropyl-β–cyclodextrin (HP-β-CD) was used as the carrier. The use of cyclodextrins derivatives (HP-β-CD for instance) is justified because β-CD can be toxic for the kidney and can show nephrotoxicity in case of parenteral administration [37]. Mammucari et al. reported that HP-β-CD and methyl-β-CD can be used for coprecipitate naproxen in the form of microparticles with an enhanced dissolution rate [36]. However, the morphology of the SAS-prepared powders drug–HP-β-CD is in most cases not satisfactory, since crystals, aggregates, coalescing particles, or big particles with holes mainly precipitated. Nevertheless, the formation of drug–CD complexes via the SAS process led to a drug dissolution rate increase. Conversely, the interesting results reached by Lee et al. [20], Franco and De Marco [35], Jia et al. [38], and Nerome et al. [39] revealed the possibility to achieve a regular and spherical morphology of composite particles using β-CD as carrier and scCO_2_ as antisolvent. In summary, β-CD, which can be safely used in the case of oral administration, seems to be a more suitable carrier for SAS precipitation compared to HP-β-CD. Aiming to mask the bitter taste of cetirizine (an antihistamine) with β-CD, Lee et al. [20] attempted to produce inclusion complexes by both freeze-drying and SAS technique. Operating at molar ratios of cetirizine:β-CD equal to 1:3, 1:2 and 1:1, large and irregular crystals precipitated via freeze-drying; whereas, regular and spherical particles were produced by the SAS technique. These results also showed the superiority of supercritical fluid-assisted technology in overcoming the main drawbacks of conventionally used techniques, even though none of the produced cetirizine–β-CD systems show any change in the antihistamine drug dissolution rate. Instead, in the study of Nerome et al. [39], the dissolution tests were not performed at all, and the formation of lycopene/β-CD inclusion complexes was claimed only by DSC analyses; i.e., because of the disappearance of the cetirizine melting peak in the DSC thermograms of SAS powders.

Considering all the previous assumptions, this paper is focused on the production of RUT–β-CD inclusion complexes by SAS process, to improve the rutin dissolution in an aqueous medium and, consequently, its bioavailability. The final purpose is to propose alternative formulations or supplements, exploiting the numerous beneficial properties of the natural active compound and improving its therapeutic efficacy.

## 2. Materials and Methods

### 2.1. Materials

β-cyclodextrin (β-CD, purity 99.9%) and rutin hydrate (RUT, purity 95%) were purchased by Sigma–Aldrich (Milan, Italy). Dimethylsulfoxide (DMSO, purity 99.5%) was bought from Carlo Erba (Cornaredo, Italy). Carbon dioxide (CO_2_, purity 99%) was supplied by Morlando Group s.r.l. (Sant’Antimo, Italy). The solubility of RUT in DMSO, which was experimentally determined at about 25 °C, is approximately 240 mg/mL.

### 2.2. SAS Apparatus and Procedure

**Figure 1:**
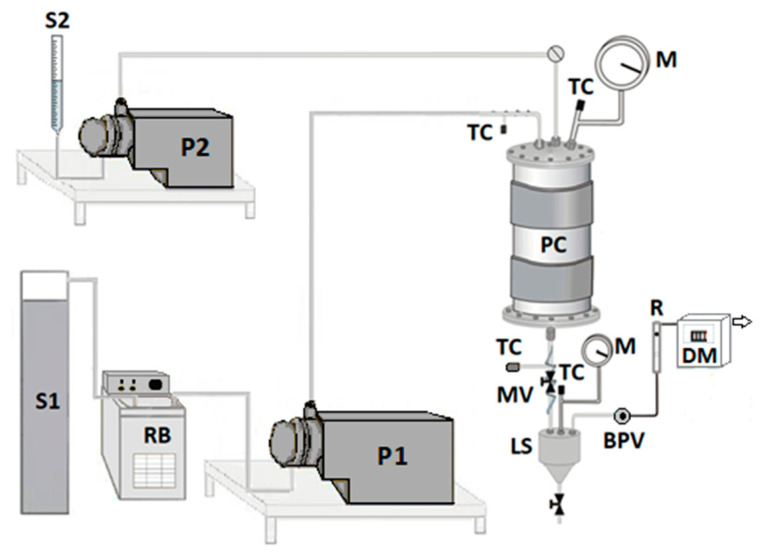
A schematic representation of the SAS laboratory plant.

The bench plant used for the SAS tests is drafted in Figure 1. Briefly, the high-pressure pump P1 allows delivering the CO_2_ (stored in reservoir S1) to the precipitation chamber (PC, internal volume of 500 cm^3^). The high-pressure pump P2 permits to feed the liquid solution (stored in the burette S2), which consists of the solutes (RUT, β-CD) solubilized in the liquid solvent (DMSO); in detail, it is injected through a 100 µm internal diameter stainless-steel nozzle. The temperature control (TC) in the PC is ensured by a controller connected with an electrically thin band. The pressure is determined by a test gauge manometer (M), whereas its regulation is assured through a micrometric valve (MV). The precipitated powder is collected at the bottom of the PC on a stainless-steel filter (size of pores of around 0.1 μm), which also allows the passage of the mixture CO_2_–DMSO. Downstream the PC, the DMSO is recovered in the separator LS, whose pressure is fixed at a lower value than that into the PC (about 2.0–2.5 MPa) through the backpressure valve BPV. The flow rate and the total quantity of delivered CO_2_ are measured at the exit by a rotameter (R) and a dry test meter (DM), respectively.

At the beginning of a typical precipitation experiment, the selected temperature and pressure are reached by pumping the CO_2_ to the PC, which is heated up in a controlled manner. Once the operating conditions are stabilized, pure DMSO is firstly injected into the PC, followed by the liquid solution, inducing the precipitation of the solutes because of the supersaturation. At the end of the solution injection, the CO_2_ lasts to flow to eliminate the solvent residues. At the end of the washing step, the PC is depressurized up to the atmospheric pressure. Finally, the precipitated powder is recovered and characterized.

### 2.3. Analytical Methods

The precipitated powders were analyzed by Field Emission Scanning Electron Microscopy (FESEM, mod. LEO 1525, Carl Zeiss SMT AG, Oberkochen, Germany) to determine the morphology and size of obtained particles. The powders, which were dispersed on a carbon tab stuck on an aluminum stub, were coated with gold-palladium (layer thickness 250 Å) using a sputter coater (mod. 108 A, Agar Scientific, Stansted, United Kingdom). For each sample, the diameters of about 1000 particles were measured from various FESEM photomicrographs, by using the Sigma Scan Pro image analysis software (release 5.0, Aspire Software International Ashburn, Ashburn, VA, USA). These data were used to determine particle size distributions (PSDs) by Microcal Origin Software (release 8.0, Microcal Software, Inc., Northampton, MA, USA).

Fourier transform infrared (FT-IR) spectroscopy (M2000 FT-IR spectrophotometer, MIDAC Co, Costa Mesa, CA, USA) was employed to record the spectra of samples in the range of wavenumbers 4000–450 cm^−1^, at a resolution of 0.5 cm^−1^ and as the average of 16 scan signals. The mixture of about 100 mg of potassium bromide (KBr) and 1 mg of each sample is compressed through a hydraulic press to prepare the disc that has to be analyzed.

A differential Scanning Calorimeter (DSC, mod. TC11, Mettler-Toledo, Inc., Columbus, OH, USA) with the MettlerSTARe system was used to perform the calorimetric analyses. A sample of the powder (5 ± 0.5 mg) is heated from 25 °C up to 250 °C (with a heating rate of 10 °C/min) using a nitrogen gas flow equal to 50 mL/min.

X-ray diffraction (XRD) patterns were recorded by an X-ray powder diffractometer (Bruker, Billerica, MA, USA). The operating conditions of the analysis are the following: Ni-filtered Cu Kα radiation, 2θ angle between 10° and 50° (with a scan rate of 0.5 s/step and a step size of 0.08°), λ = 1.54 Å.

RUT dissolution tests were performed using a UV–vis spectrophotometer (model Cary 50, Varian, Palo Alto, CA, USA), at a wavelength of 257 nm. The tests were conducted in a dissolution medium constituted by phosphate buffered saline solution (PBS) at pH 7.4. Samples containing an equivalent amount of RUT (5 mg) were suspended in 3 mL of PBS placed into a dialysis sack. Then, this sack was incubated in 300 mL of PBS, continuously stirred at 150 rpm, and heated at 37 °C. Each analysis, which was performed in triplicate, was stopped at the end of RUT release; i.e., when the plateau was reached and all RUT was released to the outer aqueous phase. A calibration curve was determined using diluted standards at five different concentrations of RUT in PBS and was used to convert the absorbance into RUT concentration. The calculated calibration curve (with a correlation coefficient R^2^ = 0.9985) had the form: y = 16.413·x, where y is the absorbance and x the RUT concentration. 

The residual solvent (DMSO) in the SAS powders was measured by coupling a headspace sampler (model 7694E, Hewlett Packard, Palo Alto, CA, USA) to a gas chromatograph equipped with a flame ionization detector (GC-FID, model 6890 GC-SYSTEM, Hewlett Packard, Agilent Technologies Mfg. Gmbh & Co., Santa Clara, CA USA). Two fused silica capillary columns connected in series by press-fit were used to separate the solvent: The first column (model Carbowax EASYSEP, Stepbios, Bologna, Italy) was connected to the detector (30 m length, 0.53 mm i.d., 1 m film thickness), whereas the second one (model Cp Sil 5CB CHROMPACK, Stepbios, Bologna, Italy) was connected to the injector (25 m length, 0.53 mm i.d., 5 m film thickness). GC conditions were: oven temperature at 160 °C for a total time equal to 8.80 min. The injector was maintained at 250 °C (split mode, ratio 5:1); helium was used as the carrier gas (2 mL/min). Head space conditions were: equilibration time, 9 min at 170 °C; pressurization time, 0.3 min; loop fill time, 0.4 min. Fifty milligrams of drug dissolved in water were placed in 20-mL vials to prepare the head space samples.

## 3. Experimental Results and Discussion

All SAS experiments were performed using DMSO as the liquid solvent. The fixed operating conditions are the following: a temperature of 40 °C and the flow rates of CO_2_ and liquid solution equal to 30 g/min and 1 mL/min, respectively. In particular, the indicated flow rates were chosen to operate with CO_2_ molar fractions approximately equal to 0.98 at the selected temperature; i.e., on the right of the Mixture Critical Point (MCP) of the binary system CO_2_–DMSO to assure the supercritical mixture conditions [40,41].

A list of the SAS tests performed at various operating conditions is reported in Table 1, in which the morphology of the obtained particles with the mean diameter (m.d.) and the standard deviation (s.d.) on a volumetric basis are indicated.

**Figure 2:**
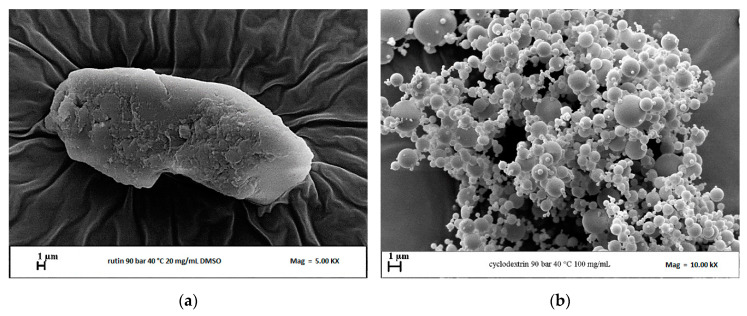
FESEM images of powders precipitated from DMSO at 9 MPa and 40 °C: (**a**) RUT (run #1); (**b**) β-CD (run #2).

Some preliminary tests were performed micronizing RUT and the polymer separately by the SAS technique. Processing RUT at 40 °C, 9 MPa with a solute concentration in DMSO equal to 20 mg/mL (run #1 in Table 1), most of the solute was extracted by the DMSO–scCO_2_ mixture. Analyzing through FESEM the small amount of RUT recovered on the filter, the presence of irregular crystals emerged (Figure 2a), indicating that it is not possible to process RUT alone by the SAS technique. If the pressure is increased at 12 and 15 MPa (not reported in the table), RUT is extracted from the DMSO–scCO_2_ mixture and no powder at all is recovered inside the precipitator. This result is confirmed in literature [42].

β-CD was micronized at 40 °C, 9 MPa, and a solute concentration in DMSO equal to 100 mg/mL (run #2). Spherical microparticles (Figure 2b) precipitated from DMSO, in agreement with the results obtained in previous work [43], in which the tendency of β-CD to precipitate in the form of microparticles at various pressures, temperatures, and concentrations in DMSO, was highlighted.

Hence, the coprecipitation of RUT with β-CD was studied to form inclusion complexes by the SAS process. In literature, it was demonstrated that it is possible to force even the morphology of active compounds that cannot be processed alone via SAS technique, like RUT, by selecting the proper polymeric carrier [30]. To obtain well-defined microparticles, the effect of various parameters, namely the concentration of solutes in DMSO, the pressure, and RUT:β-CD molar ratio, on particle morphology and size were investigated.

### 3.1. Effect of Total Concentration on RUT/β-CD Coprecipitates

**Figure 3:**
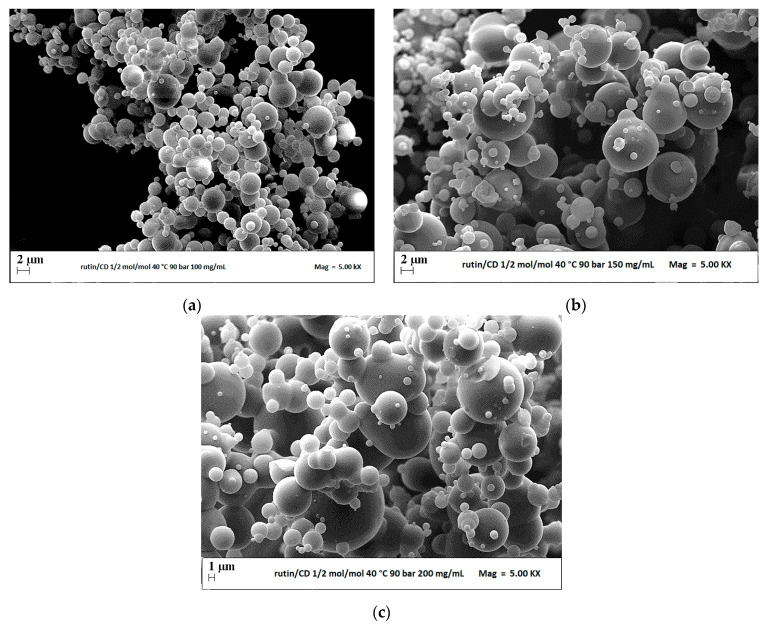
FESEM images of microparticles RUT–β-CD 1:2 mol:mol precipitated from DMSO at 40 °C, 9 MPa and (**a**) 100 mg/mL (run #3); (**b**) 150 mg/mL (run #4); (**c**) 200 mg/mL (run #5).

The first set of experiments (runs #3–5) was performed to investigate the effect of the overall concentration of solutes in DMSO, at a fixed pressure of 9 MPa and RUT:β-CD ratio equal to 1:2 mol:mol. Increasing the concentration from 100 to 200 mg/mL, well-separated and spherical microparticles were obtained in all cases, as it is possible to observe in Figure 3.

**Figure 4:**
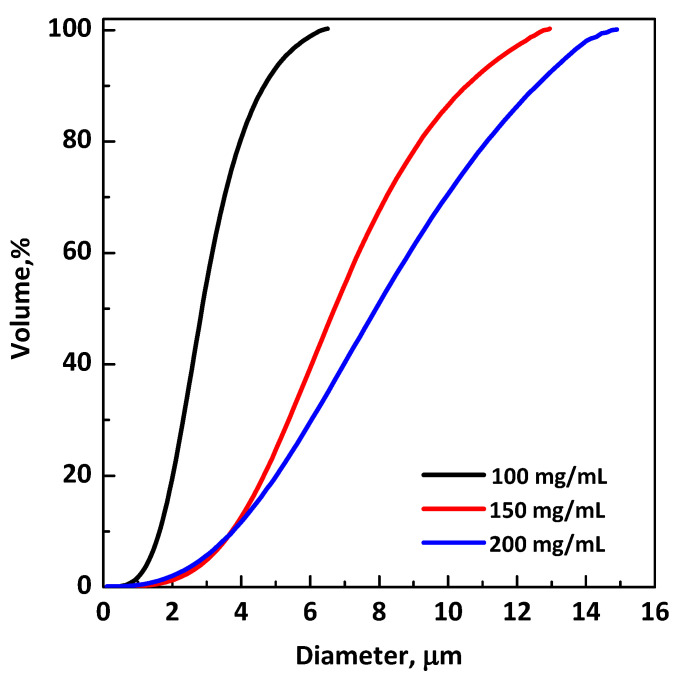
Volumetric PSDs of RUT:β-CD microparticles 1:2 mol:mol; effect of total concentration in DMSO.

The influence of the total concentration of solutes in the liquid solution is shown in Figure 4, where a comparison of the volumetric cumulative PSDs of particles precipitated at 40 °C and 9 MPa is reported. It can be noted that the dimensions of microparticles increased by increasing the concentration in DMSO.

### 3.2. Effect of Pressure on RUT/β-CD Coprecipitates

**Figure 5:**
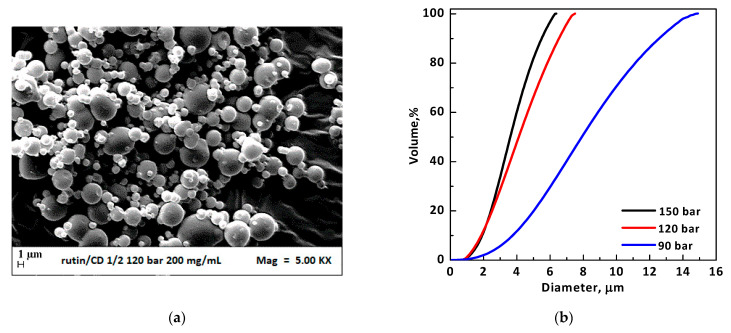
(**a**) FESEM image of RUT–β-CD 1:2 mol:mol microparticles precipitated from DMSO at 40 °C, 200 mg/mL, and 12 MPa (run #6); (**b**) volumetric PSDs of RUT–β-CD microparticles 1:2 mol:mol: effect of the operating pressure.

The subsequent experiments (runs #5–7 in Table 1) were performed at a total concentration equal to 200 mg/mL and a RUT:β-CD ratio fixed at 1:2 mol:mol. To investigate the effect of pressure on the produced particles, this process parameter was increased from 9 MPa to 15 MPa. Well-separated microparticles were obtained in correspondence of all the tested pressures (exemplificative FESEM images obtained 9 MPa and 12 MPa are reported in Figure 3a and Figure 5a, respectively). The PSDs of particles precipitated at 40 °C and 200 mg/mL are compared in Figure 5b to highlight the effect of the operating pressure. It was noted that, by decreasing the pressure, the mean particle size increased and the PSD enlarged.

### 3.3. Effect of RUT/β-CD Molar Ratio

The influence of the RUT:β-CD molar ratio was also studied because it seems to be the parameter that mostly affects the complexation process [21]. To further reduce the amount of the carrier in the composites, the RUT:β-CD ratio was gradually increased from 1:2 to 2:1 mol:mol. 

Considering that, under the conditions of test #5 (9 MPa, 40 °C, 200 mg/mL), well-defined RUT–β-CD 1:2 mol:mol microparticles were obtained; firstly the reduction of the guest:host molar ratio at 1:1 was attempted, keeping the other parameters unchanged (run #8). However, operating under these conditions, the liquid was recovered in the vessel at the end of the experiment. This outcome could be explained considering the high-pressure vapor-liquid equilibria (VLEs). At the selected temperature of 40 °C, the MCP of the binary system DMSO–scCO_2_ is located at 8.61 MPa [40]; therefore, the operating point at 9 MPa was theoretically above the MCP of the binary system solvent/antisolvent. However, the high concentration of RUT–β-CD in the liquid solution could have modified the binary system VLEs; this occurrence may induce a shift of the RUT–β-CD–DMSO–scCO_2_ quaternary system MCP towards higher pressures compared to the MCP of the solvent/antisolvent binary system [41]. Consequently, the operating point at 9 MPa could be below the MCP and located in the biphasic region in case of run #8.

**Figure 6:**
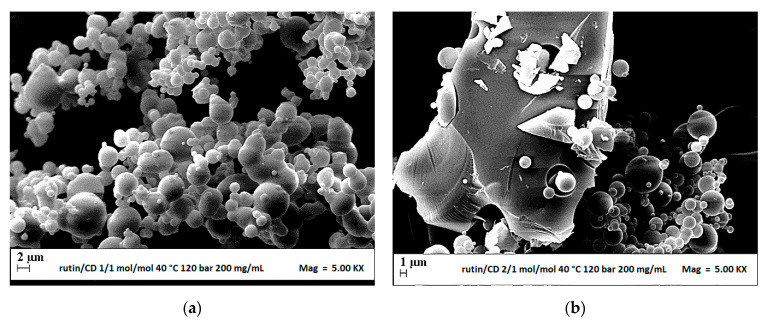
FESEM images of (**a**) microparticles RUT–β-CD 1:1 mol:mol (run #9), (**b**) microparticles and crystals at RUT–β-CD 2:1 mol:mol (run #10), precipitated from DMSO at 40 °C, 200 mg/mL and 12 MPa.

For this reason, the following test (run #9) was performed at 200 mg/mL and RUT:β-CD ratio 1:1 mol:mol, but increasing the pressure at 12 MPa to shift the operating point above the MCP of the quaternary system. Microparticles at molar ratio guest:host 1:1 (Figure 6a) were produced at 12 MPa. Given the positive result, a further increase of the RUT:β-CD ratio at 2:1 mol/mol working at 12 MPa and 200 mg/mL (run #10) was attempted. In Figure 6b, it can be observed that, in this case (run #10), large crystals and microparticles precipitated from DMSO, meaning that the coprecipitation was not effective and that the operating point was near/below the MCP. This result confirms that the presence of RUT strongly modifies the VLEs of the DMSO–CO_2_ binary system and that it is important to identify, for each ternary or quaternary system, the proper operating conditions to conduct the experiments.

### 3.4. Characterization of RUT/β-CD Samples

To verify the formation of inclusion complexes, SAS powders were characterized by different analytical techniques.

**Figure 7:**
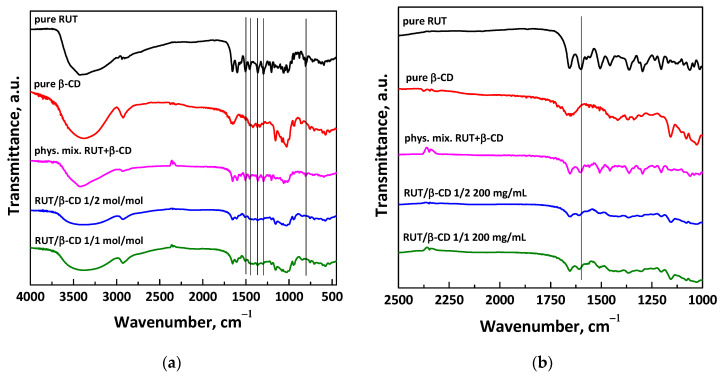
FT-IR spectra of unprocessed RUT and β-CD, physical mixture NSAID+ β-CD and SAS-processed RUT–β-CD powders at different molar ratios: (**a**) entire spectra; (**b**) enlargement of the spectra.

The FT-IR spectra of unprocessed RUT and β-CD, physical mixture RUT + β-CD (1:1 mol:mol), and SAS processed RUT–β-CD at different molar ratios are reported in Figure 7. The spectrum of pure RUT shows various characteristic absorption bands, including the stretching carbonyl group at 1654–1600 cm^−1^, that of the ether group at 1362–1169 cm^−1^, and the characteristic adsorption band of Ar at 1507 cm^−1^ [44]. The FT-IR spectrum of pure β-CD shows the absorption bands attributed to the C=O stretching vibrations of the glycosidic bond, the primary alcohol, and the cyclic alcohol at 1022 cm^−1^, 1638 cm^−1^, and 1156 cm^−1^, respectively [45]. The characteristic bands of RUT are evident in the spectrum of RUT + β-CD physical mixture unlike the spectra of SAS-processed powders RUT–β-CD both at 1:2 mol/mol and 1:1 mol:mol. The disappearance of absorption bands or the reduction in peak intensity in the FT-IR spectra of active compound–β-CD samples can be attributed to the formation of inclusion complexes [20,31,32,33,34,46], since the active compound (guest) is incorporated into the β-CD cavity (host). In addition to the partial or complete disappearance of RUT bands, like those at 1169, 1362, and 1654 cm^−1^, it is possible to note a shift of the RUT band at 1600 cm^−1^ towards slightly higher wavenumbers, as observable in the enlargement of FT-IR spectra reported in Figure 7b. According to the literature [47], this shift can be ascribed to the formation of hydrogen bonds between the carbonyl group and the hydroxyl group of RUT and β-CD, proving again the attainment of inclusion complexes.

**Figure 8:**
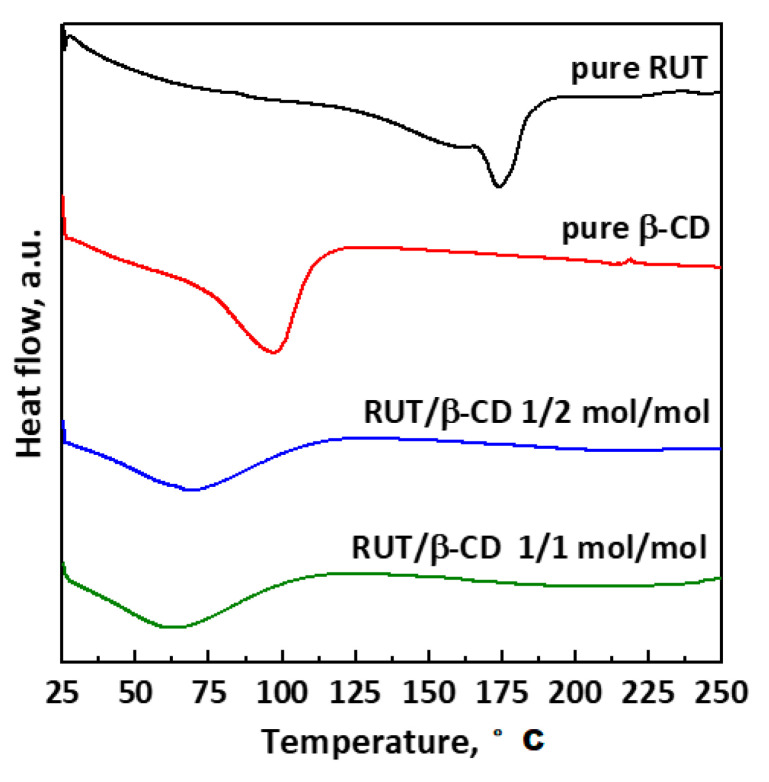
DSC thermograms of unprocessed RUT, unprocessed β-CD, and SAS-processed RUT–β-CD powders at different molar ratios.

DSC thermograms of unprocessed RUT and β-CD and SAS-processed RUT–β-CD at different molar ratios (1:2 and 1:2 mol:mol) are reported in Figure 8. The thermogram of pure RUT exhibits an endothermic peak in the range 143–190 °C, which is related to the phase transition and the molecular rearrangement of RUT; i.e., the melting point [15]. It is possible to note that this characteristic peak of rutin disappears in the thermograms of SAS-processed RUT–β-CD powders because RUT is encapsulated in the β-CD cavity thanks to the formation of weak interactions between the host and the guest [31,32,33]. Moreover, the thermogram of the pure β-CD shows a broad endothermic peak in the range 75–115 °C because of the dehydration [13]. The intensity of this peak reduces in the case of all SAS RUT–β-CD powders, due to the loss of water molecules in the β-CD cavity replaced by the hydrophobic RUT. Indeed, this replacement allows reaching a lower and more stable energy state with the formation of inclusion complexes [48]. 

**Figure 9:**
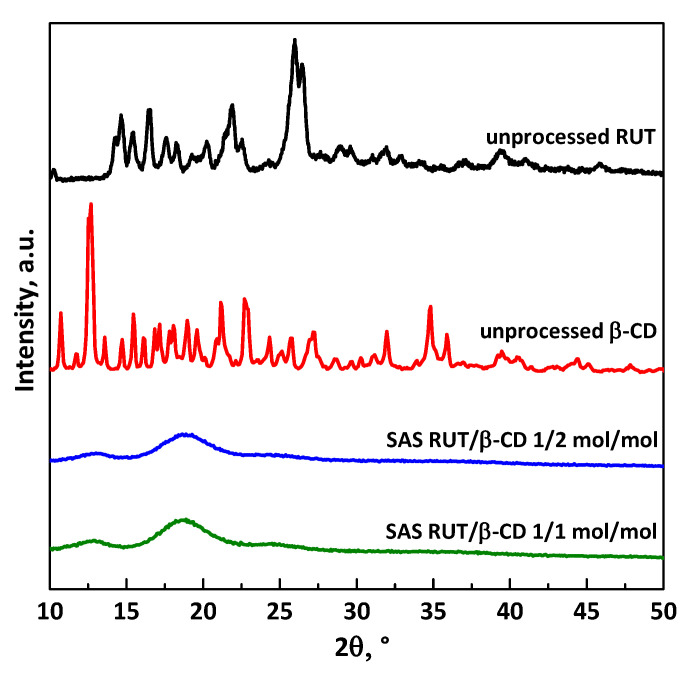
XRD patterns of unprocessed RUT and β-CD, and SAS-processed RUT–β-CD powders at different molar ratios.

XRD analyses were also performed on unprocessed RUT and β-CD, and SAS processed RUT–β-CD at different molar ratios (i.e., 172 mol:mol and 1:1 mol:mol), as reported in Figure 9. The XRD patterns of pure RUT and β-CD show the crystalline state of both the materials, whereas all SAS RUT–β-CD powders show an amorphous structure. This change in the solid-state of materials is attributed in the literature to the formation of amorphous inclusions [31]: The characteristic peaks of the active compound disappears in the XRD patterns of SAS powders because RUT molecules are incorporated and “hidden” in the cavity of the amorphous β-CD.

**Figure 10:**
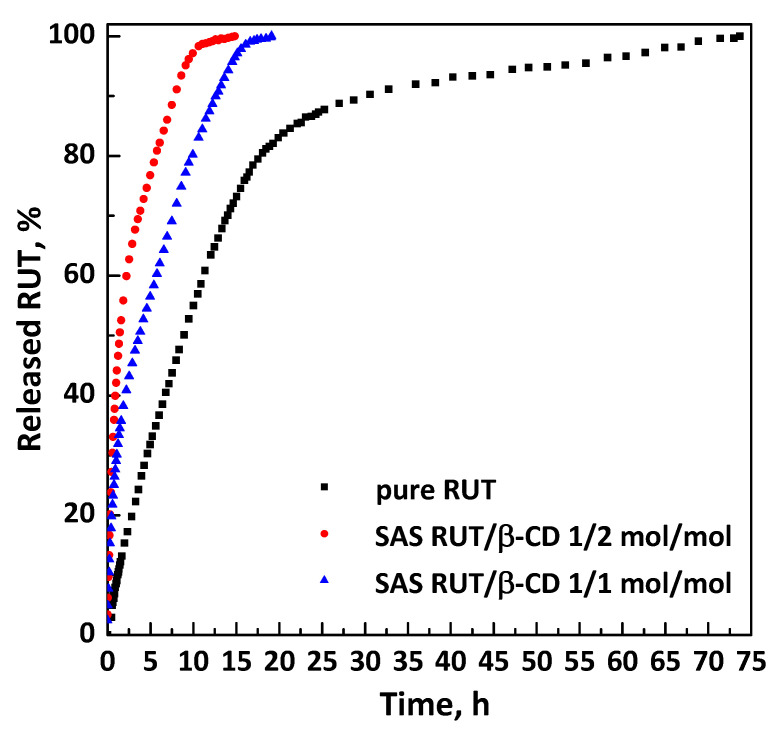
Dissolution profiles in PBS at pH 7.4 and 37 °C.

Dissolution tests (Figure 10) were performed to compare the dissolution rate of unprocessed active compound and RUT from SAS complexes at different molar ratios of RUT:β-CD (1:2 mol:mol and 1:1 mol:mol). It was observed that 90% of pure RUT dissolved in PBS in about 30.4 h, whereas it took about 7.7 and 12.7 h in the case of SAS complexes RUT–β-CD 1:2 and 1:1 mol:mol, respectively. Therefore, the dissolution rate of RUT is increased up to about 3.9 and 2.4 times with RUT–β-CD 1:2 and 1:1 mol:mol inclusion complexes, respectively.

According to the Food and Drug Administration (FDA) guidance about residual solvents in pharmaceutical (or nutraceutical) products, the maximum allowed concentration of DMSO in the final product is 5000 ppm, since it belongs to class 3 (solvents with low toxic potential) [49]. The residual DMSO content in the SAS precipitated RUT–β-CD powders was determined; the analyses revealed that the solvent residue was in the range 300–600 ppm for all the SAS samples.

It is worth noting that very satisfactory results are obtained in this study since a significant improvement of the rutin dissolution is achieved at very low active compound:β-CD ratios, which are generally difficult to reach by coprecipitating a drug with a generic polymer through SAS technique [30,50]. This means that the formation of inclusion complexes with β-CD allows reducing significantly the amount of the carrier into the coprecipitated powders, with respect to the composite microspheres generally obtained by SAS coprecipitation with other polymers. In particular, in a previous work [15], composite microparticles with a well-defined morphology containing rutin were obtained only at drug:polymer ratios equal to 1:20 and 1:10 *w*:*w* using PVP, which is currently one of the best hydrophilic carriers for SAS coprecipitation [30]. Instead, in this work, inclusion complexes with good morphology and micrometric size are obtained at RUT:β-CD ratios equal to 1:4 *w*:*w* (1:2 mol:mol) and 1:2 *w*:*w* (1:1 mol:mol). Moreover, SAS powder RUT–β-CD 1:2 *w*:*w* (1:1 mol:mol) not only has a reduced quantity of polymer while maintaining a microparticle-like morphology but shows a dissolution rate about two times faster than SAS coprecipitates RUT–PVP 1:10 *w*:*w* [15].

## 4. Conclusions and Perspectives

SAS coprecipitation revealed to be suitable to form RUT–β-CD inclusion complexes. Different parameters influenced the morphology and dimensions of the composite precipitated particles. In particular, RUT:β-CD molar ratios equal to 1:2 mol:mol and 1:1 mol:mol have to be selected for proper coprecipitation and complexation. The FT-IR, DSC, and XRD analyses demonstrated the formation of guest–host inclusion complexes at rutin:β-CD ratios equal to 1:2 and 1:1 mol/mol. Specifically, FT-IR analyses proved the formation of non-covalent interactions after the SAS process, which are typical of inclusion complexes. This led to a significant increase in the dissolution rate of RUT, which was about 3.9 and 2.4 times faster compared to pure rutin in the case of the 1:2 and 1:1 mol:mol RUT–β-CD inclusion complexes, respectively. Moreover, it was observed that the formation of inclusion complexes based on β-CD allowed to reduce significantly the amount of the carrier in SAS coprecipitated powders. These results are relevant from a pharmaceutical/nutraceutical point of view since SAS RUT–β-CD composites can be proposed as supplements that allow benefiting from all the properties of the vitamin P.

## Figures and Tables

**Table 1:** A summary of SAS experiments performed (P = pressure; C_tot_ = total concentration of solutes in DMSO; MP = microparticles; C = crystals).

#	RUT/β-CD (mol/mol)	RUT/β-CD (*w*/*w*)	P (Mpa)	C_tot_ (mg/mL)	Morphology	m.d. ± s.d. (μm)
1	1/0	1/0	9	20	C	-
2	0/1	0/1	9	100	MP	1.91 ± 0.54
3	1/2	1/4	9	100	MP	2.88 ± 0.88
4			9	150	MP	6.72 ± 1.74
5			9	200	MP	7.94 ± 2.12
6			12	200	MP	4.12 ± 1.14
7			15	200	MP	3.63 ± 1.09
8	1/1	1/2	9	200	liquid	-
9			12	200	MP	1.45 ± 0.88
10	2/1	1/1	12	200	MP + C	-

## Data Availability

All data in this study were generated by our research group, and they are included in this article.
